# Case Report: A Case of a Patient with Smith–Magenis Syndrome and Early-Onset Parkinson’s Disease

**DOI:** 10.3390/ijms25158447

**Published:** 2024-08-02

**Authors:** Tchelet Stern, Yara Hussein, Diogo Cordeiro, Hagit Sadis, Tali Garin-Shkolnik, Ronen Spiegel, Sagit Cohen, Ruth Harari, Ilana Schlesinger, Shani Stern

**Affiliations:** 1 Sagol Department of Neurobiology, University of Haifa, Haifa 3103301, Israel; 2 Center for Rare Diseases, Emek Medical Center, Afula 1834111, Israel; 3 Pediatric Department B, Emek Medical Center, Afula 1834111, Israel; 4 Nofim, Daycare, Migdal 1495000, Israel; 5 Department of Neurology, Rambam Health Care Campus, Haifa 3109601, Israel

**Keywords:** SMS, ID, RAI1, PD, neurodegenerative disease

## Abstract

Smith–Magenis Syndrome (SMS) is a rare genetic disorder, characterized by intellectual disability (ID), behavioral impairments, and sleep disturbances, as well as multiple organ anomalies in some affected individuals. The syndrome is caused by a deletion in the chromosome band around 17p11.2, including the Retinoic Acid Induced 1 (*RAI1*) gene, a multifaceted transcriptional regulator that modulates the expression of genes involved in cellular proliferation and neurodevelopment. This gene has a positive role in regulating BDNF and, importantly, affects several cell mechanisms and pathways such as the nigro-striatal pathway, which is crucial for motor function. Parkinson’s disease (PD) is one of the most common neurodegenerative diseases in older populations. It is characterized by various physical symptoms including tremors, loss of balance, bradykinesia, and a stooping posture. We present a case study of a patient diagnosed with both SMS and early-onset PD (at the age of 49). The association between both conditions is as yet ambiguous. Genome-wide association studies (GWAS) implicate an association between the RAI1 gene and PD. Similarly, the co-existence of both SMS and PD in the patient suggests a possible association between *RAI1* copy number variations (CNVs) and PD, further indicating that RAI1 has strong implications for PD pathogenesis. Our results suggest that RAI1 CNVs and the pathophysiology of PD may be related, underscoring the need for further research in this field. Therefore, caregivers of SMS patients should pay careful attention to the possibility of their patients developing EOPD and should consider starting treatment for PD as soon as the first symptoms appear.

## 1. Introduction

Smith–Magenis syndrome (SMS; OMIM #182290) is a rare genetic multisystemic developmental disorder [1] with a birth prevalence of approximately 1:25,000 [2]. The syndrome was first observed by Ann Smith et al. in 1986, when they described nine unrelated patients as having a new rare genetic disease [3] and showing a chromosomal deletion in the short arm of chromosome 17 in a portion of the 17p11.2 band, causing haploinsufficiency of the retinoic acid-induced 1 (*RAI1*) gene [3,4]. Eight of the described patients, who had partial deletion, presented similar symptoms: behavioral problems, intellectual disability, aggressiveness, sleep disturbances, psychomotor and growth issues, delayed speech, brachycephaly, prognathism, a hoarse voice, and hearing loss. The remaining patient, who had a complete deletion of band 17p11.2, was more extremely affected, showing severe facial, genitourinary, cardiac, and skeleton malformations, and died at the young age of six months [3]. Most of the affected individuals do carry a 17p11.2 deletion, while others who lack 17p11.2 deletions carry various heterozygous nucleotide changes in the *RAI1* gene consisting of both nonsense and missense mutations, as well as deletions affecting one or more nucleotides [5,6,7]. The *RAI1* gene is expressed in many tissues [8] and is highly expressed in neurons in the brain during development and adult life [9]. It codes for the retinoic acid-induced 1 protein [9]. It was first discovered in 1995 as a primary regulator of neuronal and glial differentiation [9], intricately involved in neurodevelopmental processes, behavioral modulation, and the orchestration of circadian rhythms, notably impacting the sleep–wake cycle [4]; bone and skeletal development; renal and cardiovascular function; and cellular growth and survival, reflecting some of the clinical characteristics observed in SMS [10]. Interestingly, gene expression analysis of mouse models haploinsufficient in *RAI1* revealed reduced levels of brain-derived neurotrophic factor (BDNF), while overexpression of *RAI1* resulted in increased BDNF expression, indicating a positive role for RAI1 in the regulation of BDNF [11].

Previous research has shown that alterations in retinoic acid signaling may contribute to the pathogenesis of Parkinson’s disease (PD) [12]. Initial evidence suggests that the development of the nigro-striatal pathway, crucial for motor function, is regulated by retinoid receptors, which are highly expressed in the striatum. Mutant mice lacking these retinoid receptors show impaired dopaminergic development and motor function, indicating the receptors’ potential role in PD pathogenesis [12,13,14]. Additionally, when nanoparticles loaded with retinoic acid were injected into PD mouse models, a significant reduction in dopamine neuron degeneration in the substantia nigra pars compacta was observed in these models [15].

PD is the second most common neurodegenerative disease after Alzheimer’s disease [16], and was introduced as a neurological syndrome in 1817 by Dr. James Parkinson, who described it as a “shaking palsy” [17]. Early descriptions of the disease can be found in Indian and Chinese sources dating back to 1000 BC [18,19]. Additionally, Sylvius de Le Boe also wrote about “rest tremor” in 1680 [20], and Sauvages wrote about it in 1768 [21]. Since then, much more knowledge has been obtained about the pathophysiology of PD. The hallmark of the disease is often considered to be Lewy bodies’ neuronal inclusions in brain areas, caused mostly by the aggregation of α-synuclein species [21,22], although not all patients have these inclusions [23]. Patients exhibit massive neuronal cell loss in the substantia nigra pars compacta, which is associated with deficits in their motor control [24]. Cellular models for PD have recently been constructed using animal models [25,26] and induced pluripotent stem cell (iPSC) models [27,28,29], showing synaptic dysfunction and dysregulation of extracellular matrix-related pathways [30,31].

PD is an age-related disorder, and it is more common in the elderly [32]. Late onset of PD (LOPD) is defined when the patient is over 50 years of age during the onset. The incidence of PD increases with age, with a prevalence of approximately 1% among individuals over the age of 65 [33,34]. Early-onset Parkinson’s disease (EOPD) is usually treated and is defined when symptoms of PD initiate before the age of 50 [35,36]; EOPD usually progresses more rapidly compared to LOPD [34].

Having a family history of PD increases the risk of the disease by three to four times [37], which supports genetic involvement in the disease [38]. Although many genes have been implicated in PD’s pathophysiology in the last few years, the disease is still considered sporadic in the majority of patients [39]. The associated genes can be of low penetrance, for example, *CGH1*, *GAK*, *MAPT*, and some *SNCA* variants. There can also be genes of moderate penetrance such as *LRRK2* variants and *GBA1*, or genes with high penetrance such as *PINK1*, *PRRK7*, and *PRKN* [37,40,41,42].

Some of these mutations cause alpha-synuclein aggregation, which may form Lewy bodies, disrupting normal cellular signaling pathways. Mutations in the *LRRK2* gene, for example, disrupt various cellular functions, including neurite outgrowth, cytoskeletal maintenance, vesicle trafficking, autophagic protein degradation, and immune system regulation [43]. Mutations in the *GBA1* gene, such as the E326K variant, have been linked to decreased enzymatic activity and the resulting buildup of glycolipids in cells, causing cellular dysfunction and neurodegeneration [44].

In the last couple of decades, genome-wide association studies (GWAS) have provided the opportunity to find associated genes in addition to causative genes. Interestingly, some studies have associated the *RAI1* gene with PD [45,46,47].

## 2. Case Presentation

A 54-year-old patient has been diagnosed with SMS and EOPD, and was diagnosed with the latter five years ago, at the age of 49. The patient’s parents were not related; the gestation of the patient was normal, and so was the delivery; and he weighed 2.6 kg at birth. His mother developed LOPD when she was 72 years old, with mild symptoms and a slow progression of the disease, controlled with a low dosage of levodopa/carbidopa. He has two healthy female siblings. His grandfather (from his father’s side) had late-onset, rapidly progressing Alzheimer’s disease.

A fluorescence in situ hybridization test (FISH) was performed, targeting the 17p11.2 region, that detected a small deletion that included the *RAI1* gene in only one of the chromosomes, thus supporting the diagnosis of SMS. The karyotype was normal.

The patient’s parents noticed developmental delays in speech and motor function along with sleep disturbances before he reached one year of age—a typical symptom of the condition. He suffered from behavioral issues during childhood and adolescence, such as aggression, and was treated with selective serotonin reuptake inhibitors (SSRIs) and antipsychotic drugs in addition to non-steroidal anti-inflammatory drugs (NSAIDs) for treating his self-injurious behavior. He was diagnosed with autism spectrum disorder (ASD) associated with attention deficit hyperactivity disorder (ADHD) and ID. During his childhood, he suffered from onychotillomania (pulling nails), scratching wounds, polyembolokoilamania (insertion of objects into bodily orifices), aggressiveness, and sleep disturbances, and at an older age he experienced hearing issues, all of which is typical for SMS patients. He had cryptorchidism (undescended testicle) that had been surgically corrected, and he also had myopia.

At the age of 36 years, his height was 1.68 m (30th percentile) and he weighed 57 kg (5th percentile), with a head circumference of 57.5 cm (90th percentile). He had moderate to severe hair loss; a widow’s peak; thick eyebrows; supraorbital ridging; hypoplasia of the maxilla; prognathism; relatively short mild pectus excavatum; mild thoracic kyphosis and mild lumbar lordosis; freckles on his back; 5th finger clinodactyly in one hand; skin with signs of premature aging and scars; multiple wounds; and dysplastic nails in his toes. During the examination, he showed many stereotypical hand movements.

Five years ago, at the age of 49, the patient started suffering from falls and tremors that were assumed to be due to epilepsy, although several electroencephalogram (EEG) tests were performed, and there was no trace of convulsive disorders. Therefore, he was given valproic acid. There was no improvement in his symptoms. A head computer tomography (CT) examination showed calcifications in the left frontoparietal regions with sclerosis that had been unchanged since 2014.

He slowly developed postural instability and bradykinesia, together with increased tremors—typical motor symptoms in EOPD—despite having normal blood pressure.

At the age of 53, the patient exhibited rest tremors, muscle rigidity, and gait disturbances. He could get up from a chair and walk briskly while leaning slightly forward, with much instability. At the time, he was taking paroxetine, valproic acid, and nabumetone regularly because he was injuring himself repetitively. He was taking vitamin D and other vitamins due to deficiencies. He was also taking ursodiol for cirrhosis and portal hypertension. He was then diagnosed with EOPD. Valproic acid was discontinued and treatment with levodopa/carbidopa (750 mg daily) was initiated. He responded well to this treatment, with a reduction in tremors and falls.

He is currently residing in an institution for intellectually impaired patients, where he receives good care. He currently uses half a pill of Levodopa/Carbidopa 250 mg three times a day (t.i.d); paroxetine 20 mg once a day (q.d); lansoprazole 30 mg (q.d) in the morning before breakfast; and vitamin D 400 UI (q.d), ferrous iron 100 mg, folic acid 400 mcg (q.d), and amoxicillin 500 mg twice a day (b.i.d) when he has open wounds.

He had a good response to PD treatment with levodopa/carbidopa, with a reduction in tremors and some improvement in walking and muscle rigidity. However, a few months ago, he started having minor urinary incontinence that was screened by a urologist without any findings, and this is possibly a new PD symptom.

The patient does not use a dental plate even though it is needed, due to pulling out of the dental plate by the patient and, therefore, his diet is made of mashed foods and liquids. He now weighs 50.1 kg, his height is 165 cm, and he has a BMI of 18.4. His diet is hyperproteic, with about 1900 Kcal and around 90 g of protein daily.

Every six months, he goes through a clinical evaluation. His laboratory exams are good except for minor normocytic anemia (hemoglobin: 11–12 g/%) and elevated liver enzymes, alanine aminotransferase (ALT: 189 U/L), and aspartate aminotransferase (AST: 170 U/L), which have been stable in previous exams. His liver function, albumin, gamma-glutamyltransferase (GGT), bilirubin, coagulation, serum proteins, and platelets are normal.

The patient does not have any visceral abnormalities, although he has developed hepatic cirrhosis with a negative work-up. It was concluded that this is most likely medication-induced. Because of his liver problem, he underwent a liver biopsy that showed cirrhosis with no other findings or causes. His abdominal ultrasound showed cirrhosis with hepatosplenomegaly and portal hypertension. His gallbladder is normal, and his endoscopies have not shown any esophageal varices. During a routine evaluation of his cardiovascular system, a minor pericardial effusion was detected, so every six months he undergoes a transthoracic echocardiogram.

**Figure 1:**
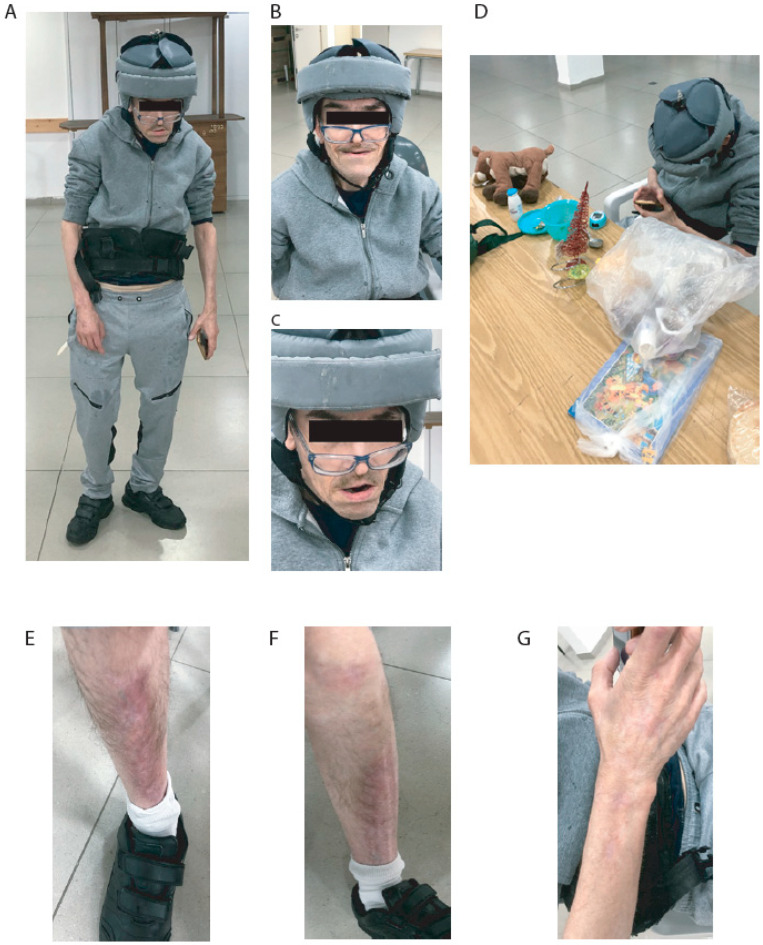
Pictures of patient. (**A**) Posture and whole body. (**B**) Face while smiling. (**C**) Face. (**D**) Playing with toys and cell phone. (**E**) Right leg scars. (**F**) Left leg scars. (**G**) Right arm scars.

The nurses of the institution say that he has no aggressive behavior toward colleagues. Sometimes, he injures himself by scratching, pulling nails, and inserting objects into bodily orifices. Currently, the patient has SMS features, scars on his legs and arms from previous wounds, and some stereotypical mouth and hand movements. The patient has occasional falls and, therefore, uses a helmet for protection. Additionally, he uses a vest to correct his posture due to his spine deformities. During the meeting, he was polite, asked questions, and ate yogurt and avocado. He had some toys with him and received some presents. After a brief talk, he was more entertained by the toys and games on a cellular phone than by the visitors. His appearance is shown in Figure 1.

## 3. Discussion

Smith–Magenis Syndrome is caused by a de novo deletion of the RAI1 gene on chromosome band 17p11.2, leading to haploinsufficiency. This genetic anomaly results in a range of phenotypic features, including ID, behavioral problems, and various skeletal, craniofacial, and visceral abnormalities [5]. The SMS phenotype is congruent with neurological and behavioral symptoms; craniofacial and skeletal anomalies; ocular malformations; otolaryngology abnormalities; obesity; and dental, kidney, or cardiovascular abnormalities. Notable clinical features in previous reports were ID, sleep disturbances, motor and speech delays, onychotillomania, polyembolokoilamania, other self-injurious behaviors, chronic ear infections, hearing loss, hoarse voice, brachycephaly, prognathism, myopia, and strabismus [48,49]. This responsible gene is widely expressed in various tissues, is highly prominent in neurons, and has been identified as a key regulator of neuronal and glial differentiation.

Retinoic acid (RA) signaling is vital in apoptosis, differentiation, and cellular proliferation. Improper signaling is associated with neuropsychiatric disorders such as PD, ASD, depression, schizophrenia, and bipolar disorder, as well as skeletal and limb defects [50,51]. Recent research indicates that a common genetic variant regulates the mRNA expression of RAI1 in the human brain within a binding site for retinoic acid RXR/RAR receptors, and these changes in mRNA expression during early brain development may contribute to the development of these disorders [50]. Altered retinoic acid signaling that has been reported in SMS patients has been linked to PD pathogenesis, along with striatal retinoid receptors essential for nigro-striatal pathway development and motor function. Mutant mice lacking these receptors display impaired dopaminergic development and motor function, indicating the receptors’ involvement in both conditions. Additionally, sleep disturbance and alteration of the circadian rhythm, including changes in the periods of melatonin secretion, are common features of SMS and can serve as early signs of the disorder [3,49]. Both SMS and PD patients can have alterations in their *CLOCK* genes (e.g., *CLOCK*, *RAI1*, *NR1D1*, *BMAL*, and *PER1*, among others) [5,42]. Patients with other rare disorders including Huntington’s disease, Rett syndrome, Angelman syndrome, and Down syndrome are reported to have an increased likelihood of developing Parkinson’s disease compared to the general population [52,53,54,55] due to shared muscle tone abnormalities, such as hypo- and hypertonia, that affect movement control and coordination, as well as tremors and bradykinesia.

Recently, novel approaches to understanding the pathophysiology of neuropsychiatric disorders have been developed using induced pluripotent stem cells (iPSCs) for several brain disorders [56,57]. In a recent study, Stern et al. [27] used dopaminergic (DA) neurons derived from iPSCs of PD patients and controls. Known mutations of PD and sporadic PD have demonstrated that electrophysiological changes in DA neurons and common pathways occur in sporadic and mutant PD cell lines, and these affected pathways identified by gene expression analyses can be considered for future research. The electrophysiological changes include a reduction in synaptic activity that was also observed in ASD models [57,58,59,60]. However, different types of neurons were differentiated (cortical and hippocampal for ASD and DA neurons for the PD study).

## 4. Conclusions

This case report presents a 54-year-old patient with SMS caused by a heterozygous deletion of the *RAI1* gene on chromosome 17p11.2, who also developed EOPD which was diagnosed five years ago. The patient exhibits the typical features of SMS, including intellectual disability, behavioral problems, sleep disturbances, craniofacial abnormalities, and skeletal deformities. Additionally, he has developed motor symptoms characteristic of EOPD, such as tremors, bradykinesia, muscle rigidity, and gait disturbances, which have responded well to levodopa/carbidopa treatment.

The co-occurrence of SMS and EOPD in this patient is noteworthy, as recent studies have suggested a potential association between the *RAI1* gene and Parkinson’s disease pathogenesis. The *RAI1* gene plays a crucial role in neurodevelopmental processes, behavioral modulation, and circadian rhythms, which are implicated in both SMS and PD, and reduced RAI1 levels lead to decreased BDNF, highlighting RAI1’s role in BDNF regulation. Additionally, alterations in retinoic acid signaling and retinoid receptors, which are crucial for nigro-striatal pathway development, have been linked to PD, with mutant mice that lack these receptors showing impaired dopaminergic development and motor function. To our knowledge, this is the first case report of a patient with SMS and EOPD, highlighting a novel potential link between these conditions. This case supports previous reports of an association between the *RAI1* gene and PD [46]. Suggesting that mutations such as deletions of the *RAI1* gene may be causative of PD is coherent with reported data about the potential effect of RAI1 protein on the development of the nigro-striatal pathway. Despite the lack of knowledge about the overlap of SMS and PD, this study could be the first to shed light on a possible causative role of RAI1 mutations in PD, and could lead to the evaluation of more underdiagnosed patients. This report may enhance awareness and encourage additional research, possibly focusing on the mechanisms and the neurophysiology phenotypes of human-derived neurons from SMS patients. This is crucial for targeting the condition’s symptoms, potentially facilitating earlier EOPD diagnosis.

## Data Availability

The data presented in this case report are available on request from the corresponding author. The data are not publicly available due to privacy protection.
